# The forgotten people: Hepatitis B virus (HBV) infection as a priority for the inclusion health agenda

**DOI:** 10.7554/eLife.81070

**Published:** 2023-02-09

**Authors:** Emily Martyn, Sarah Eisen, Nicky Longley, Philippa Harris, Julian Surey, James Norman, Michael Brown, Binta Sultan, Tongai G Maponga, Collins Iwuji, Stuart Flanagan, Indrajit Ghosh, Alistair Story, Philippa C Matthews

**Affiliations:** 1 The Francis Crick Institute London United Kingdom; 2 London School of Hygiene & Tropical Medicine London United Kingdom; 3 Hospital for Tropical Diseases, Division of Infection, University College London Hospitals NHS Foundation Trust London United Kingdom; 4 Department of Infectious Diseases, University College London Hospitals NHS Foundation Trust London United Kingdom; 5 Find & Treat Service, Division of Infection, University College London Hospitals NHS Foundation Trust London United Kingdom; 6 Institute of Global Health, University College London London United Kingdom; 7 Universidad Autonoma de Madrid, Ciudad Universitaria de Cantoblanco Madrid Spain; 8 Mortimer Market Centre, Central and North London NHS Foundation Trust London United Kingdom; 9 Stellenbosch University, Faculty of Medicine and Health Sciences Tygerberg South Africa; 10 Department of Global Health, Brighton and Sussex Medical School, University of Sussex Brighton United Kingdom; 11 Africa Health Research Institute Durban, KwaZulu-Natal South Africa; 12 Collaborative Centre for Inclusion Health, University College London London United Kingdom; 13 Division of Infection and Immunity, University College London London United Kingdom; University of California, San Francisco United States; McGill University Canada

**Keywords:** hepatitis b virus, inclusion health, sustainable development goals, refugee health, health inequality, homelessness, elimination, public health

## Abstract

Hepatitis B virus (HBV) infection represents a significant global health threat, accounting for 300 million chronic infections and up to 1 million deaths each year. HBV disproportionately affects people who are under-served by health systems due to social exclusion, and can further amplify inequities through its impact on physical and mental health, relationship with stigma and discrimination, and economic costs. The ‘inclusion health’ agenda focuses on excluded and vulnerable populations, who often experience barriers to accessing healthcare, and are under-represented by research, resources, interventions, advocacy, and policy. In this article, we assimilate evidence to establish HBV on the inclusion health agenda, and consider how this view can inform provision of better approaches to diagnosis, treatment, and prevention. We suggest approaches to redress the unmet need for HBV interventions among excluded populations as an imperative to progress the global goal for the elimination of viral hepatitis as a public health threat.

## Introduction

**Figure 1.**
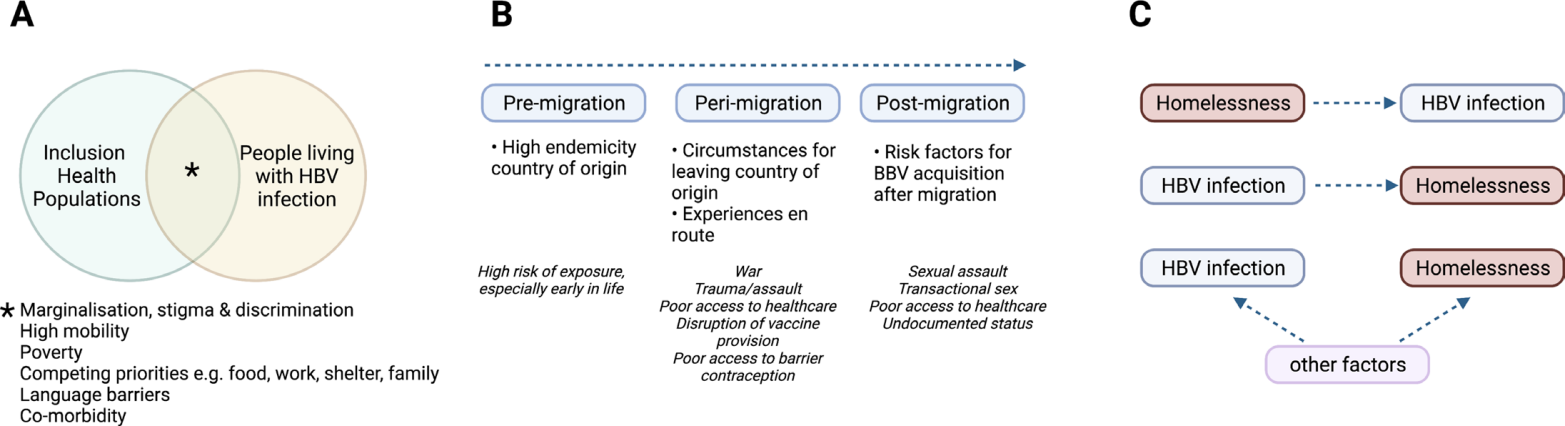
Characteristics of HBV in inclusion health populations. (**A**) Illustration of the overlapping characteristics that may be present among different inclusion populations and people living with HBV infection. (**B**) Relationship between migrancy and asylum-seeking status as a risk factor for HBV infection. (**C**) Representation of complex relationship between HBV infection and PEH, where other factors include for example injecting drug use, transactional sex, mental illness (Freeland et al., 2021; Ly et al., 2021a). HBV – hepatitis B virus; PEH - People experiencing homelessness. Figure created in BioRender.com with a licence to publish.

Hepatitis B virus (HBV) infection is estimated to account for 300 million chronic infections and 1 million deaths each year (World Health Organization, 2021). The global burden of HBV infection is unevenly distributed, with particularly high prevalence in some populations in Africa and South East Asia (Cooke et al., 2019). Likewise, HBV is not experienced equally across society, with a disproportionate prevalence and impact on marginalised and deprived populations, whose needs are poorly met by existing healthcare research, services, interventions and policies (O’Hara et al., 2017; Figure 1A). The ‘inclusion health’ agenda focuses on these populations, setting out to identify and address health and social inequities, for example barriers to accessing mainstream healthcare services (Royal College of Physicians, 2022; Aldridge et al., 2018). These groups include, but are not limited to, migrants in vulnerable situations, people experiencing homelessness (PEH), individuals with substance use disorders, commercial sex workers (CSW), incarcerated individuals, and Roma and Traveller communities (Royal College of Physicians, 2022).

Viral hepatitis is recognised as a global health priority within the United Nations Sustainable Development Goals 2030 (SDG30). These underpin ambitious targets including 90% HBV vaccine coverage, reducing new chronic infections and new infections by 90% and attributable mortality by 65% (Cooke et al., 2019). Vaccination against HBV, including universal birth dose administration, is a key elimination strategy and is estimated to have prevented 310 million cases of HBV infection between 1990 and 2020 (Cooke et al., 2019, de Villiers et al., 2021). However, birth dose vaccine implementation has been slow in reaching the most vulnerable populations, while a three-dose vaccine schedule remains a challenge (de Villiers et al., 2021). Lack of education and awareness, inadequate living conditions, lack of access to healthcare services, and poverty and stigma among marginalised populations create a permissive environment for HBV transmission. Chronic HBV infection usually remains asymptomatic until late complications (liver fibrosis, cirrhosis, and hepatocellular carcinoma (HCC)). Silent infection, in combination with barriers to testing in the most affected populations, explains why only an estimated 10% of those living with HBV infection are diagnosed (O’Hara et al., 2017).

There is no cure for HBV, but antivirals (nucleos(t)ide analogue agents) can suppress viral replication. Based on international treatment guidelines, only a minority of those with chronic infection are eligible for treatment, and a small proportion of these can actually access consistent therapy (McNaughton et al., 2021). Risk stratification requires consistent surveillance (including clinical review, imaging and laboratory tests), but this can feel burdensome and irrelevant for some people living with asymptomatic infection and/or those who do not meet treatment criteria (Mofokeng, 2021, Adjei et al., 2019). Moreover, HBV receives only a fraction of global research funding allocated to major infectious diseases. For example, in 2022, projected US funding for HIV research is 20-fold greater than for HBV, despite the lower number of people living with HIV infection (National institutes of health, 2022). Therefore, HBV remains a major public health threat, with 1.5 million new infections each year and more annual deaths than malaria or HIV (O’Hara et al., 2017, Graber-Stiehl, 2018).

An advocate with lived experience of HBV infection described the HBV community as ‘the forgotten people’, a description that is also pertinent to inclusion health populations (Matthews et al., 2022a, Aldridge et al., 2018). These exclusions may lead to an increased risk of HBV infection, are amplified by HBV infection, and lead to greater morbidity and mortality as a result of inadequate access to care. In resource-limited settings, where the burden of HBV infection is greatest, the inequities are amplified.

We have collated evidence for the impact of HBV in some of these marginalised populations globally, assimilating data and identifying knowledge gaps in order to inform the HBV agenda in inclusion health. Addressing the complex intersection of social exclusion and HBV infection is essential to support progress towards global elimination goals.

### HBV infection in inclusion health populations

In this section, we review data for different populations who are frequently marginalised and under-served by healthcare provision. The characteristics, experiences and needs of these groups are diverse, but also have features in common, and approaches should be integrated as far as possible.

#### Migrants

By the end of 2020, there were 281 million international migrants, equivalent to 3.6% of the world’s population (International organisation for migration, 2021). A significant proportion (approaching 90 million) are displaced due to conflict, political instability, and natural disasters. In Europe, more than half of 49 million migrants born outside of the European Economic Area came from nations where HBV endemicity is either intermediate (2–8%) or high (>8%), explaining the higher prevalence of HBV infection in migrants (5%), and asylum seekers (10%) compared to the general population (1%) in Europe (European Centre for Disease Prevention and Control, 2016; Kim et al., 2021; Table 1). Descriptive studies from Italy and the UK provide some insight into HBV infection among migrants and unaccompanied asylum-seeking children (Table 2; Prestileo et al., 2022, Colucci et al., 2022; Mazzitelli et al., 2021; Armitage et al., 2022; Williams et al., 2020a, World Health Organization, 2002), reporting risks of infection that apply before, during and after relocation (Figure 1). In some countries, including the UK, asylum seekers may be moved hundreds of miles away at short notice, disrupting any continuity of care (Farrant et al., 2022; World Health Organization, 2018b).

**Table 1.** Top 20 origins of international migrants in 2020 (millions), HBV prevalence and progress towards SDG 30 goals for elimination of HBV as a public health threat. Data - United Nations Department of Economic and Social Affairs, Population Division (2020b). International Migrant Stock 2020. https://www.un.org/development/desa/pd/content/international-migrant-stock; The Polaris Observatory, CDA Foundation; https://cdafound.org/polaris-countries-compare/. HBV – Hepatitis B virus. SDG – Sustainable Development Goal.

Country of Origin	Number of International Migrants (millions)	HBV Prevalence*	90% Diagnosed†	80% Treated†	65% Reduction in Mortality†	Reduced Prevalence in 5 year olds†
India	17.9	3%	2051	2051	2051	2032
Mexico	11.2	0%	2051	2051	2051	2015
Russia	10.8	1%	2051	2051	2051	2015
China	10.5	6%	2051	2051	2051	2021
Syria	8.5	6%	2051	2051	2051	2051
Bangladesh	7.4	5%	2051	2051	2051	2043
Pakistan	6.3	1%	2042	2051	2051	2036
Ukraine	6.1	1%	2051	2051	2051	2030
Philippines	6.1	10%	2051	2051	2051	2051
Afghanistan	5.9	3%	2051	2051	2051	2045
Venezuela	5.4	1%	2051	2051	2051	2031
Poland	4.8	1%	2051	2051	2051	2015
United Kingdom	4.7	1%	2051	2051	2051	2020
Indonesia	4.6	7%	2051	2051	2051	2051
Kazakhstan	4.2	4%	2051	2051	2051	2027
Palestine	4.0	2%	2051	2051	2051	2015
Romania	4.0	3%	2051	2051	2051	2025
Germany	3.9	0%	2039	2051	2051	2015
Myanmar	3.7	8%	2051	2051	2051	2051
Egypt	3.6	1%	2051	2051	2051	2018

* Green -Low HBV prevalence (<2%); Amber - intermediate HBV prevalence (2–8%); Red - high HBV (prevalence >8%).

† Green - HBV SDG reached before 2030; Amber - SDG reached 2031–50; Red - SDG reached >2050.

**Table 2.** Key review and study observations pertinent to HBV infection among inclusion health populations.

Inclusion health population	Citation	Study type	Country	Key observations
PEH, PWID, Incarcerated individuals, sex workers	Aldridge et al., 2018	Systematic Review and Meta-analysis	High-income countries	All-cause mortality significantly increased for inclusion health populations studied: Female SMR 11.86 (95% CI 10.42–13.3; I^2^ 91.5%) Male SMR 7.88 (95% CI 6.40–9.37; I^2^ 98.1%) High SMR for infectious diseases: 11.43 (95% CI 6.91–15.94; I^2^ 97%) High prevalence of hepatitis B Sex workers contributed only 4.2% datapoints, compared to people with substance use disorders contributing 42.1%
PWID, PEH, Previous incarceration	Taylor et al., 2019	Cross-sectional	UK	346 participants Level of self-declared HBV vaccination 52.3% Being female associated with lower HBV vaccine uptake (OR 2.37, 95% CI 1.23–4.57, p=0.01) Intravenous drug use protective against incomplete vaccination (OR 0.23, 95% CI 0.08–0.62, p=0.004) 2 or more risk factors associated with protection against incomplete vaccination (OR 0.19, 95% CI 0.09–0.39, p<0.001)
Migrants	Prestileo et al., 2022	Cross-sectional	Italy	Screening migrants for HBV and other blood borne viruses on arrival to Italy is acceptable to target population (>95% uptake) Risk of HBV infection was associated with: Female sex (aOR 2.47,95% CI 1.90–3.20, p=0.003) Physical and/or sexual violence on migration journey (aOR 2.24,95% CI 1.87–3.55,p<0.001)
	Colucci et al., 2022	Cross-sectional	Italy	Increased risk of BBV acquisition persisted after arrival in Italy, possibly due to living conditions, sex work, lack of access to healthcare and social support
	Mazzitelli et al., 2021	Cross-sectional	Italy	Migrants appeared to have severe chronic HBV disease requiring intervention: >2/3 of migrants with CHB had moderate/ severe liver disease and were eligible for HBV treatment >10% diagnosed with HBV had liver cirrhosis <50% of migrants diagnosed with CHB were retained in care at 1 year
	Armitage et al., 2022	Cross-sectional	UK	UASC in London (n=101) 16% female, median age 16 (range 14–17) Physical assault/abuse reported in 67% 13% disclosed sexual assault/abuse (38% of females) Mental health symptoms in 77% 6% prevalence HBV infection
	Williams et al., 2020a	Cross-sectional	UK	UASC in London (n=252) 19 countries (majority from Afghanistan, Eritrea, Albania) 4.8% prevalence HBV infection (highest prevalence from Sudan and Afghanistan) Many co-infections (TB, Schistosomiasis most common)
	Eborall et al., 2020	Qualitative	UK	Very low knowledge and low personal perceived risk of acquiring HBV among migrants Majority of migrants with positive views around screening for infectious diseases including HBV. Reservations included: Concerns over results impacting immigration and/or asylum applications Concerns over delay receiving the results Language barriers Stigma
	Tasa et al., 2021	Retrospective cohort	Finland	62 pregnancies, undocumented women 4 women received no antenatal care, 3 denied antenatal care 71% received less that the recommended number of antenatal vistits 3% HBV seroprevalence, significantly higher than all pregnant women (p=0.007) 2/3rds attended first prenatal visit in 2^nd^ or 3^rd^ trimester
	Bierhoff et al., 2021	Mixed-methods	Thailand	Migrant pregnant women, northern Thailand 757 knowledge and attitude studies: Low knowledge about HBV transmission, infection or vaccination (28% correct response) Qualitative analysis found counselling should: Use appropriate language, tailored to local health literacy level, provide pertinent information, be repeated over antenatal period, ensure privacy
PEH	Ly et al., 2021a	Narrative review	Global	HBV seroprevalence higher than in non-PEH Studies have associated HBV past infection in PEH with: older age, MSM, insertive anal sex, Injecting drug use, alcohol use
	Al Shakarchi et al., 2020	Systematic review and meta-analysis	Global	Compared to non-homeless individuals, homeless individuals: More likely to have cardiovascular disease (pooler OR 2.96, 95% CI 2.80–3.13 l p<0.001, I=99.1%) More likely to have hypertension (pooled OR 1.38–1.75, p=0.007) Higher cardiovascular mortality (age-standardised SMR range 2.6–6.4)
PWID	Degenhardt et al., 2017	Multistage systematic review	Global	Estimated global seroprevalence active HBV of 9% (95% UI 5.1–13.2%) Highest seroprevalence in: East and SE Asia (20%) Azerbaijan, Egypt, Cote d’Ivoire, Lithuania, Belarus, Czech Republic (>10%) 21.7% (15.8–27.9) recently (within past year) experienced homelessness/ unstable housing 57.9% (50.5–65.2) with history of incarceration
People who misuse alcohol	Magri et al., 2020	Systematic review and meta-analysis	Global	12,204 alcohol users, mostly men Estimated global seroprevalence of active HBV infection was 20% (95% CI 19–20) Substantial heterogeneity between studies (I^2^=96.7%)
Incarcerated people	Dolan et al., 2016	Systematic review and meta-analysis	Global	Global chronic HBV seroprevalence among incarcerated individuals estimated at 4.8% Regions with the highest HBsAg seroprevalence were: West and central Africa (23.5%, 95% CI 19.8–27.5) Eastern Europe and central Asia (10.4%; 95% CI 1.9–24.6%)
	Nakitanda et al., 2021	Descriptive analysis	Europe	Data from WHO Prison’s European Database and ECDC hepatitis prevalence database HBsAg seroprevalence ranged from 0% in maximum-security prison in the UK to 25% in two Bulgarian juvenile detention centres Universal HBV screening on opt-out basis available in 31% of reporting countries Condoms and lubricants offered free of charge in 46% and 15% of reporting countries, respectively Universal HBV vaccination available in 85% of reporting countries
	Kamarulzaman et al., 2016	Narrative review	Global	25% PWID initiate drug use in prison Risk factors for HBV: sharing needles, sharing toothbrushes, unsterile tattooing and body piercing
	Dana et al., 2013	Cross-sectional	Iran	Prevalence of HBV among incarcerated PWID associated with: Multiple incarceration (OR 1.43, 95% CI 1.01–2.02) Total duration of incarceration (OR 2.70 95% CI 1.94–3.74)
Sex workers	Schuelter-Trevisol et al., 2013	Cross-sectional	Brazil	3 cities, southern Brazil 147 SWs (Male 4.5%, female 91.2%, transgender 4.3%) 3.4% HBsAg positive Baseline prevalence <2% in Brazil
	Miranda et al., 2021	Cross-sectional	Brazil	4 cities, northern Brazil 365 FSWs 3.0% HBsAg positive Use of illicit drugs most strongly associated with exposure to HBV (OR 44.1, 95% CI 12.7–68.6)
	Matos et al., 2017	Cross-sectional	Brazil	One city, mid-western Brazil 402 FSWs 1.6% HBsAg positive Only 28% serological evidence of HBV vaccination Exposure to HBV associated with: Age >40 (OR 3.5, 95% CI 1.5–7.9, p<0.001) Being in education <4 yrs (OR 3.2, 95% CI 1.4–7.4, p<0.009) Being single (OR 2.0, 95% CI 1.1–3.8, p<0.028) Meeting clients on the street (OR 2.5, 95% CI 1.4–4.4, p<0.003)
	Leuridan et al., 2005	Cross-sectional	Belgium	129 MSWs 3.3% HBsAg positive Only 9.1% anti-HBs (i.e. evidence of HBV vaccination)
	Mak et al., 2003	Cross-sectional	Belgium	1096 SWs (97.8% female) 0.6% HBsAg positive Only 7% anti-HBs (i.g. evidence of previous HBV vaccination)
	Dos Ramos Farías et al., 2011	Cross-sectional	Argentina	273 trans female sex workers (TSW), 114 MSW HBV exposure 40% TSW, 22% MSW Total HbSAg prevalence 1.6%
	Todd et al., 2010	Cross-sectional	Afghanistan	HBV infection associated with: >= 12 clients monthly (OR 3.30, 95% CI 1.46–7.47) Ever using drugs (OR 1.77, 95% CI1.55–2.02) or alcohol (OR 2.96, 95% CI 2.15–4.07) Having children (OR 1.52, 95% CI 1.41–1.64)
	Jeal and Salisbury, 2004	Cross-sectional	UK	24% vaccinated against HBV Only 30% booked in first trimester and attended all antenatal appointments 13% received no antenatal care until admitted Barriers: waiting times, fear of judgement
	Bitty-Anderson et al., 2021	Cross-sectional	Togo	1036 female sex workers HbsAg prevalence 9.9% HBV infection associated with: Recruitment out of the capital city (aOR 6.63; 95% CI 2.51–13.4-, *P*<0.001) Never using condoms for vaginal intervourse (OR 3.14; 95% CI 1.02–8.71)
Roma and Traveller populations	Macejova et al., 2020	Cross-sectional	Slovakia	452 Roma people screened Increased HBsAg positivity compared to age-matched general population (RR 4.47, 95% CI 2.36–8.42; *P*<0.001)
	Gregory et al., 2014	Cross-sectional	UK	1345 Slovak-Roma patients seen in dedicated primary care clinic 9.4% HBsAg positivity (compared to 1% general UK population) Median number of people in each household 7 (range 1–21 people)
	Veselíny et al., 2014	Cross-sectional	Slovakia	452 Roma people screened, risk factors assessed by questionnaire/interview Participants who were HBsAg positive had a higher median age compared to those with no evidence of HBV exposure (35.2, vs 30.7, *P*=0.028) A higher proportion of male participants were HBsAg positive, compared to those with no evidence of HBV exposure (51% vs 31%, *P*=0.005) A higher proportion of incarcerated people, and people with tattoos, had HBV exposure (HBc IgG positive) compared to those with no history of exposure (HBc IgG negative) (14% vs 6%, p 0.016 and 44% vs 32%, p 0.035, respectively)
Indigenous Populations	Davies et al., 2019	Cross-sectional	Australia	35,633 individuals tested in Northern Territory between 2007–2011 HBsAg positivity was 3·40% (95%CI 3·19–3·61), being higher in Indigenous (6·08% [5·65%–6·53%]) than non-Indigenous (1·56% [1·38%–1·76%]) Australians, *P*<0·0001
	Qama et al., 2021	Retrospective cohort	Australia	100 790 individuals were tested (33.4% Indigenous) between 1991 and 2011 (26.1% of the 2011 NT population) In 2011, the proportion of HBV positive individuals in the NT was 3.17% (5.22% in Indigenous populations) compared to previous 2011 estimates of 1.70% (3.70% in Indigenous populations) Evidence of suboptimal vaccine efficacy by breakthrough anti-HBc positivity in vaccinated individuals was demonstrated in 3.1% of the vaccinated cohort, of which 86.4% identified as Indigenous (HR 1.17)
	Einsiedel et al., 2013	Retrospective cohort	Australia	558 indigenous and 55 non-indigenous community residence of central Australia HBsAg more common in indigenous compared to non-indigenous (12.9% vs 6.7%) Other infections and non-communicable diseases more common in indigenous than non-indigenous population
	Osiowy et al., 2013	Narrative review	USA, Canada, Greenland	High prevalence of HBV among indigenous populations (e.g. Inuit of Greenland 3–29% HBsAg positivity, Alsaka Native and Canadian Far North 3–20% depending on community investigated), before introduction of HBV vaccine Variety of genotype observed which may alter natural history of disease Genotype B6 (now known as B5) unique to this region, reported to have a more benign disease course
	Russell et al., 2019	Systematic review	Latin America	Reviewed 62 studies from 12 countries High endemicity (>8%) of hepatitis B was found in some indigenous peoples in Mexico (Huichol) (9.4%), Venezuela (Yanomami: 14.3%; Japreira: 29.5%) and among Afro-descendant quilombola populations in Brazil (Frechal: 12.5%; Furnas do Dionísio: 8.4% in 2008, 9.2% in 2003)

SMR Standardised Mortality Ratio; aOR adjusted odds ratio; OR odds ratio; RR relative risk; CI confidence interval; HBV hepatitis B virus; UI Uncertainty Interval; MSM men who have sex with men; PEH people experiencing homelessness, PWID people who inject drugs; UASC unaccompanied asylum-seeking children; HBsAg Hepatitis B surface antigen (active HBV infection); HBc Hepatitis B core antibody.

#### People experiencing homelessness

Approximately 150 million people worldwide were considered homeless in 2020 (Chamie, 2017). People experiencing homelessness (PEH) endure extreme social exclusion and have substantially increased mortality from any cause compared to a socially deprived population (Table 2; Aldridge et al., 2018). Seroprevalence surveys, although of varying size and quality, report higher HBV seroprevalence among PEH compared to the housed population (Ly et al., 2021a). Homelessness may be a risk factor for HBV infection or vice versa, while there are also shared risk factors which include associations with place of birth, older age, sexual partners with a history of hepatitis, and injecting drug or alcohol use (Ly et al., 2021a, Ly et al., 2021b; Figure 1C). Low HBV vaccination uptake among PEH may relate to either never being offered the vaccine, or being unable to access follow-up doses (Ly et al., 2021b, Taylor et al., 2019).

Among PEH, fear of judgement and stigma relating to homelessness can lead to distrust of healthcare workers and services, basic needs (food, clothing and shelter) are prioritised over long-term healthcare needs, and additional barriers are common (lack of insurance, transport costs, and long waiting times in unfamiliar environments) (Crone et al., 2022; Becker and Foli, 2022; Paisi et al., 2022). High prevalence of co-morbid conditions compounds social exclusion, for example severe mental illness or substance use disorders (Shepherd-Banigan, 2021).

#### People who misuse drugs and alcohol

It is estimated that there are 15.6 million People Who Inject Drugs (PWID) between the ages of 15–64 years worldwide (Degenhardt et al., 2017), while an estimated 283 million people have alcohol use disorders (World Health Organization, 2018a). HBV infection disproportionately affects people who misuse drugs and alcohol (Table 2; Degenhardt et al., 2017; Magri et al., 2020). Alcohol use is associated with increased high-risk sexual activity, drug use, and sharing injecting equipment, and therefore may increase risk of HBV transmission (Arasteh et al., 2008), while alcohol acts synergistically with HBV infection to accelerate liver damage (Gitto et al., 2009; Li et al., 2019).

Perceived lack of need, competing priorities (e.g. avoiding opioid withdrawal), discrimination, stigma and difficulty in navigating health systems are reasons that PWID and people with alcohol use disorder may avoid seeking care (Motavalli et al., 2021; Zewdu et al., 2019; Costa et al., 2020). Inadequate funding for harm reduction and addiction programmes is a barrier to HBV prevention. Criminalisation and imprisonment of PWID exacerbates transmission and creates further barriers to consistent HBV care provision (O’Keefe et al., 2017). Finally, a fifth of PWID have experienced recent homelessness (within 1 year) and over two-thirds have a history of incarceration (Degenhardt et al., 2017; Rashti et al., 2020). This intersection of overlapping risks further amplifies HBV infection and increases the challenges for healthcare.

#### Incarcerated people

At the end of 2021, the worldwide prison population was at least 10.77 million (Fair and Walmsley, 2021). The majority of published HBV literature representing prisons consists of small cross sectional seroprevalence studies, which do not reflect the dynamic nature of prison populations (Wirtz et al., 2018; Dolan et al., 2016). Nevertheless, HBV is over-represented in incarcerated individuals, with an estimated global HBsAg prevalence of 4.8% (Dolan et al., 2016; Nakitanda et al., 2021).

Prisons create a complex intersection of individual, social and environmental challenges, and have been described as a ‘concentration mechanism’ leading to amplification and dissemination of infectious diseases (Kamarulzaman et al., 2016), particularly when injecting drug use and sex work are criminalised. 25% of PWID report initiating drug use in prisons and incarceration is associated with a doubled risk of re-initiating injecting drug use (Genberg et al., 2015; Kamarulzaman et al., 2016). Sharing contaminated needles for injecting drugs, sharing tooth brushes, unsterile tattooing, body piercing and high-risk sexual activity also represent potential routes of HBV transmission within prisons (Kamarulzaman et al., 2016; Moazen et al., 2018; Kowo et al., 2021; Harawa et al., 2010). This risk continues beyond prison; the immediate period following release is associated with high risk of heightened sexual behaviours, and drug/alcohol use (Kamarulzaman et al., 2016; Dolan et al., 2016; Binswanger et al., 2007). Frequency and duration of incarceration are also positively associated with prevalence of HBV infection (Dana et al., 2013). Given the overlap of this population and many other HBV risk factors, this is a particularly important target group in which HBV interventions need to be carefully focused, both during and after incarceration periods.

#### People who engage in transactional sex

This group is highly heterogenous, representing commercial sex workers (CSW) but also a wider group of individuals practising intermittent transactional sex (in exchange for goods, drugs, accommodation etc), overlapping with PWID and PEH populations (Table 2). Sex workers are underrepresented in the literature and robust data are lacking (Aldridge et al., 2018). HBV prevalence varies between countries and sex worker populations; cross-sectional data from Brazil suggest regional differences in HBV seroprevalence (Schuelter-Trevisol et al., 2013; Miranda et al., 2021; Matos et al., 2017), and higher risk groups include male sex workers and trans-female sex workers (Leuridan et al., 2005; Dos Ramos Farías et al., 2011; Mak et al., 2003). Older age, marital status, having children, fewer years in education, drug and alcohol use, meeting clients on the street and high numbers of clients are associated with HBV exposure among CSWs (Miranda et al., 2021; Matos et al., 2017; Todd et al., 2010).

A study of street-based female sex workers (FSW) in the UK found that most did not disclose their work to a primary care practitioner, and in pregnancy, 13% sought antenatal services for the first time whilst in labour, reducing opportunities to prevent mother to child transmission of HBV. Barriers to care for FSW include waiting times, fear of judgement and travelling long distances for healthcare (Jeal and Salisbury, 2004; Mc Grath-Lone et al., 2014).

#### Roma and Traveller communities

These communities encompass diverse distinct cultural and ethnic groups, also including travelling show people and boat dwellers. In Europe, these populations number 10–12 million, but this is likely to be an underestimate due to high mobility and poor data capture (Council of Europe, 2020). These communities experiences extreme health disparities, with a 10-year lower life expectancy than the settled population in the UK (Equality and Human Rights Commission, 2017). HBV is not well studied among these populations, but in the UK a study of Roma people found a HBV surface antigen (HBsAg) prevalence of 9.4% and in Eastern Slovakia, Roma community members were four times more likely to be HBsAg-positive compared to the age-matched general population (Macejova et al., 2020; Gregory et al., 2014). Risk factors associated with HBV infection included being male, older age, tattoos, and previous imprisonment (Veselíny et al., 2014).

Mainstream healthcare services are not inclusive towards the lifestyle and culture of travelling communities, with barriers arising through discrimination, alongside lack of fixed address and low levels of literacy (McFadden et al., 2018). Belief in God’s will, prioritising work and family commitments over personal health, mistrust in healthcare services and everyday stigma likely contribute to underdiagnosis, undertreatment and lack of preventative measures for HBV infection (Prestileo et al., 2022).

#### Indigenous communities

Indigenous populations, a global term referring to people with historical ties to a land prior to colonisation, are disproportionately affected by HBV (Osiowy et al., 2013; Russell et al., 2019; Howell et al., 2019). For example, Aboriginal Australians have a higher HBV prevalence, higher rate of liver disease, and poorer outcomes, compared to non-Indigenous Australians (Plackett, 2022; Qama et al., 2021; Wigg et al., 2021). A variety of factors may account for this disparity, including reduced healthcare access due to remote location, social inequalities and multi-morbidity (Plackett, 2022; Einsiedel et al., 2013). HBV genotype may also be an important consideration; genotype C4 in Aboriginal Australians is associated with aggressive disease progression, however this impact is yet to be unpicked from the effects of social determinants of health (Davies et al., 2019; Plackett, 2022). In addition, there is some evidence that current HBV vaccine, which is designed against genotype A, may have reduced efficacy against C4 (Qama et al., 2021). Although culturally and geographically distinct, indigenous populations across the globe face an increased burden and worse outcomes from HBV, and are poorly represented by existing data. Enhanced research, allocation of resources, services, educations and intervention is required to reduce inequities.

### Sex and gender as risk factors for HBV infection

Within inclusion health populations, evidence suggests that females with HBV fare worse than males (Table 2). For example, HBV infection was twice as likely in female migrants in Italy, and a higher proportion of female migrants and unaccompanied asylum-seeking children report a history of sexual assault/abuse which represents a significant HBV transmission risk (Prestileo et al., 2022, Armitage et al., 2022). Being female was associated with lower HBV vaccination uptake in PEH, PWID and people with a history of incarceration in the UK (Taylor et al., 2019).

It could be argued that HBV presents a ‘double jeopardy’ in females, given the risk of both individual disease and mother-to-child transmission. A Finnish study found that HBV prevalence in pregnancy was significantly higher in undocumented migrants than in the general population. Most pregnant migrants entered antenatal care late, and either received inadequate care or none at all (Tasa et al., 2021). Education and awareness are also lacking among women in some inclusion health populations: pregnant migrant women in Thailand had very low levels of HBV knowledge (Bierhoff et al., 2021), and despite a high prevalence of HBV (8%), more than 90% of FSW in Mumbai had not heard of HBV infection (United Way Mumbai, 2017).

This female disadvantage observed in inclusion health populations with HBV is reflected in the dramatically increased all-cause mortality in females compared to males across the wider inclusion health population, with female standardised mortality ratio (SMR) 11.86 (95% CI 10.42–13.3; I^2^ 91.5%) vs. Male SMR 7.88 (95% CI 6.40–9.37; I^2^ 98.1%) (Aldridge et al., 2018).

These observations within inclusion health populations contrast with observations from the wider population, which suggest males have worse HBV outcomes than females, with higher rates of exposure, chronicity and complications (Brown et al., 2022). Further work is needed to determine whether this sexual dimorphism is truly reversed in HBV within inclusion health populations, and highlights the importance of considering HBV within the sociodemographic context of inclusion health populations as this may affect risk stratification and targeted elimination strategies.

### HBV and the Inclusion Health Agenda: A global perspective

The majority of existing research on inclusion health and HBV is biased towards high-income settings such as Europe and North America (Table 2). Gathering relevant data in some high endemicity countries, including countries in the WHO African, South-East Asian and Western Pacific regions is made more difficult due to criminalisation and/or stigmatisation of inclusion health populations for example CSW, PWID and LGBTQ+ communities. Depending on the sampling methods, available data suggest intermediate to high HBV prevalence in CSWs, PWID, and incarcerated people in African populations (Bitty-Anderson et al., 2021; Degenhardt et al., 2017; Dolan et al., 2016; Scheibe et al., 2020). Fear of discrimination by healthcare workers, possible legal consequences and refusal of services are just some of the barriers deterring inclusion health populations from seeking care and remaining in longer term follow up (Duby et al., 2018). Increased difficulties collecting relevant data, heightened exclusion from healthcare services and increased HBV prevalence, mean it is of even greater importance to prioritise HBV in the inclusion health agenda globally, particularly in Africa where HBV research, advocacy, representation and investment have been neglected.

### Enhancing HBV within the Inclusion Health Agenda: The next steps

In this section, we consider how the current evidence informs policy and practical strategies to advance prevention, diagnosis, clinical care and treatment for people living with HBV infection in groups who have been under-served to date.

#### Close the data gap

There are clear SDG30 goals for HBV, but it is impossible to determine the nature and magnitude of the challenge, benchmark progress, or deploy appropriate interventions without accurate epidemiological data. European Centre for Disease Prevention and Control report major data gaps, now exacerbated by the Covid-19 pandemic, with the worst impact in high-burden (often low income) settings, hampering progress towards elimination (Pley et al., 2021). Good quality HBV data for inclusion health populations are lacking, mostly reporting from small, independent, cross sectional seroprevalence studies (summarised in Table 2). Unified, transparent data collection will require increased awareness, advocacy, funding and research, with coordination between organisations (e.g. World Health Organization, United States Centers for Disease Control and Prevention, European Centre for Disease Prevention and Control) to help standardise and strengthen approaches. Crucially, evidence is needed to underpin solution-based methods rather than simply describing problems (Luchenski et al., 2018; Siersbaek et al., 2021).

#### Active case finding

Active targeted screening efforts are an important step towards HBV elimination (European Centre for Disease Prevention and Control, 2021; Lemoine et al., 2015). Availability of point-of-care hepatitis B surface antigen tests enable community-based diagnosis, although global efforts are needed to prioritise regular supply in resource-constrained settings (Lemoine et al., 2015). For migrants, community-based testing strategies are effective, with highest uptake in programmes with endorsement from the local community (European Centre for Disease Prevention and Control, 2018; Robotin and George, 2014). Outreach screening is equally important in other inclusion health populations, for example, PEH and PWID (Box 1). Such outreach screening services have been shown to be feasible and acceptable (O’Carroll et al., 2017, Carr et al., 2014), and can also be incorporated into other services (e.g. drug harm reduction programmes).

Box 1. **Case studies of inclusion health interventions.**

1. RESPOND (Farrant et al., 2022): RESPOND is a team from University College London Hospitals in the London borough of Camden, providing rapid access community based integrated screening and care planning for asylum seekers. It was formed to accommodate the needs of the rising number of people seeking asylum in temporary accommodation at the beginning of the Covid-19 pandemic. The aim is to provide a multi-disciplinary, comprehensive assessment and formulate an integrated migrant health plan, an electronic document that outlines the key issues for each family member. This document remains with the family, and is copied to the primary healthcare provider, thus removing the need for repeated assessments after each short notice relocation.

2. Find & Treat (University College London Hospitals NHS Foundation Trust, 2013): University College London Hospitals Find & Treat are a specialist outreach team working alongside over 200 NHS and third sector front-line services to tackle TB and blood borne viruses among PEH, PWID, migrants in vulnerable situations or people who have been in prison. The team is multidisciplinary, consisting of people with lived experience working as peer advocates, nurse specialists, social and outreach workers, radiographers, and doctors with primary/secondary care expertise. They use state of the art point of care diagnostics within outreach vans to help overcome barriers in access to care among socially excluded populations.

3. ‘Gypsy & Traveller Exchange’ (Leeds gate, 2022): An example of a successful programme of raising awareness is this programme, led by community members and working in partnership with local services, supported by funding from charitable organisations and local government. Their strategy is to identify community members (or ‘gatekeepers’) who are trusted to facilitate communication with healthcare services, lead cultural awareness training for healthcare workers and provide community outreach and dedicated services for Roma and Traveller populations (McFadden et al., 2018; Condon et al., 2019; Carr et al., 2014). Similar approaches could be successful in other populations to provide education and support.

4. ‘One Stop Liver Shop’ (Hla et al., 2020): The Aboriginal population in the Northern Territory has the highest prevalence of HBV in Australia (6.08% compared to the Non-Indigenous population 1.56%). The ‘One Stop Liver Shop’ was developed iteratively with a very remote Aboriginal community (>500 km from the nearest tertiary hospital in Darwin). It is made up of a specialist doctor, community based Aboriginal Health Practitioner, a sonographer and clinical nurse specialist. They use a portable ultrasound scan, a FibroSan for transient elastography and a mobile device to use the ‘Hep B Story App’, a mobile application using the relevant local language. Visits occur 4 times per year. Due to this initiative, 88% of those aware of their diagnosis were engaged in care and 16% were on treatment.

All pregnant women should be screened for HBV, and healthcare professionals should consider opportunistically offering HBV screening in high-risk groups accessing services (World Health Organization, 2020; Centers for Disease Control and Prevention, 2022). There should also be a move to integrate routine hepatitis screening into high-risk healthcare settings, for example TB, sexual health and HIV services. The European Centre for Disease Prevention and Control (ECDC) recommends universal screening in emergency departments in areas where HBV prevalence exceeds 2%, however, evidence suggests that universal emergency department screening is cost effective even at lower prevalence (>0.25%) and therefore the United Kingdom is currently implementing this policy (Williams et al., 2020b, Nebbia et al., 2022).

Multiple studies have demonstrated cost-effectiveness of HBV screening in a variety of different scenarios, including community-based outreach and low prevalence settings (Su et al., 2022, Xiao et al., 2020, Toy et al., 2022, Nayagam et al., 2016, Tordrup et al., 2020, Hutton et al., 2022, Wright et al., 2018). This is largely due to averting high costs associated with decompensated cirrhosis, HCC and liver transplantation.

#### Rethinking prevention strategies

A safe, effective vaccine against HBV has been available since 1982, and remains a key tool for the elimination of viral hepatitis (World Health Organization, 2017). The WHO has recommended a universal birth dose HBV vaccination since 2009, irrespective of the mother’s serological status, but coverage was still only 42% globally and 17% in the WHO African Region by 2021 (World Health Organization, 2022a). Modelling suggests that scaling up of a timely birth dose vaccine to >90% by 2030 would result in immediate reduction of chronic HBV incidence, and 710,000 fewer deaths in those born between 2020–2030, globally (de Villiers et al., 2021). This strategy would be a particularly important public health intervention for future inclusion health populations, in whom catch-up three-dose adult vaccination is challenging and may not be cost-effective.

While catch-up adult vaccination is not advocated at a general population level, opportunities for providing vaccination to high-risk adults must still be explored and developed, optimising approaches to deliver a three-dose schedule alongside education, proactive antenatal and sexual healthcare, and other health interventions (Box 2).

Box 2. **Example methods of increasing HBV vaccination coverage among high-risk groups**

1. Universal HBV vaccination in prisons in Scotland (Palmateer et al., 2018).
  - Following prison-related outbreaks of acute HBV among PWID, HBV vaccination for all prisoners was introduced in 1999.
  - Among recent-onset PWID in Glasgow, vaccine uptake increased from 16% in 1993 to 59% in 2009–2014 (p<0.001).
  - HBsAg prevalence 0.3% among people who commenced injecting drugs in the decade since universal vaccination induced, compared to 9% globally.
  - Vaccination is associated with 40% reduced odds of ever having HBV (aOR = 0.6, 95% CI 0.37–0.97).
2. HBV-COMSAVA (Community Screening and Vaccination in Africans) Study, Barcelona, Spain (Picchio et al., 2021).
  - Pop-up clinics at west African migrant faith-based and community organisations.
  - Individuals offered a rapid HBsAg diagnostic test and a dried blood sample for analysing HBV viral load and co-infection with hepatitis D virus.
  - If HBsAg +, same day referral to specialist with expedited referral process which did not require prior appointment.
  - If susceptible, offered HBV vaccine in situ at return appointment (‘return-date slip’ reminder given at first appointment).
  - 70% were susceptible to HBV, 74% returned to receive results and 86% of those who returned accepted vaccination.
3. Sex-worker outreach, Ghent, Belgium (Mak et al., 2003)
  - A medical doctor and social nurse visited the workplaces of sex-workers, discussed professional risks, handed out prevention materials and offered free STI screening (Chlamydia, gonorrhoea, syphilis, HIV), a cervical smear and free HBV vaccination.
  - Psychological and legal programs related to sex work could also be discussed and anonymity guaranteed.
  - Over two-thirds of susceptible sex-workers completed an accelerated HBV vaccination schedule (over 2 months), compared to less than half if given over 6 months.
  - Only 7% were vaccinated in existing services.

Given the slow and heterogenous uptake of birth dose vaccination, and challenges associated with catch-up vaccination of inclusion health populations, other prevention strategies need urgent consideration. For example, the ‘Test and Treat’ strategy which aims to suppress the reservoir of virus in the population (McNaughton et al., 2019). According to current guidelines, only 25–35% of adults with CHB are eligible for treatment, leaving the majority at risk of both longer term complications (relevant to the individual) and the potential for transmission (relevant to populations) (McNaughton et al., 2021). HBV/HIV co-infected individuals may have a treatment advantage, reflecting the benefits of greater access to early and consistent antiviral treatment compared to mono-infected individuals (Maponga et al., 2020). Potential risks should be considered: toxicity and side effects, requirement for monitoring, potential for drug resistance, limited gains from treating well people and the cost of drug and potential diversion of healthcare resources (McNaughton et al., 2021). Further research is required to assess the efficacy, feasibility and acceptability of wider treatment roll out.

#### Designing inclusive HBV healthcare

**Figure 2.**
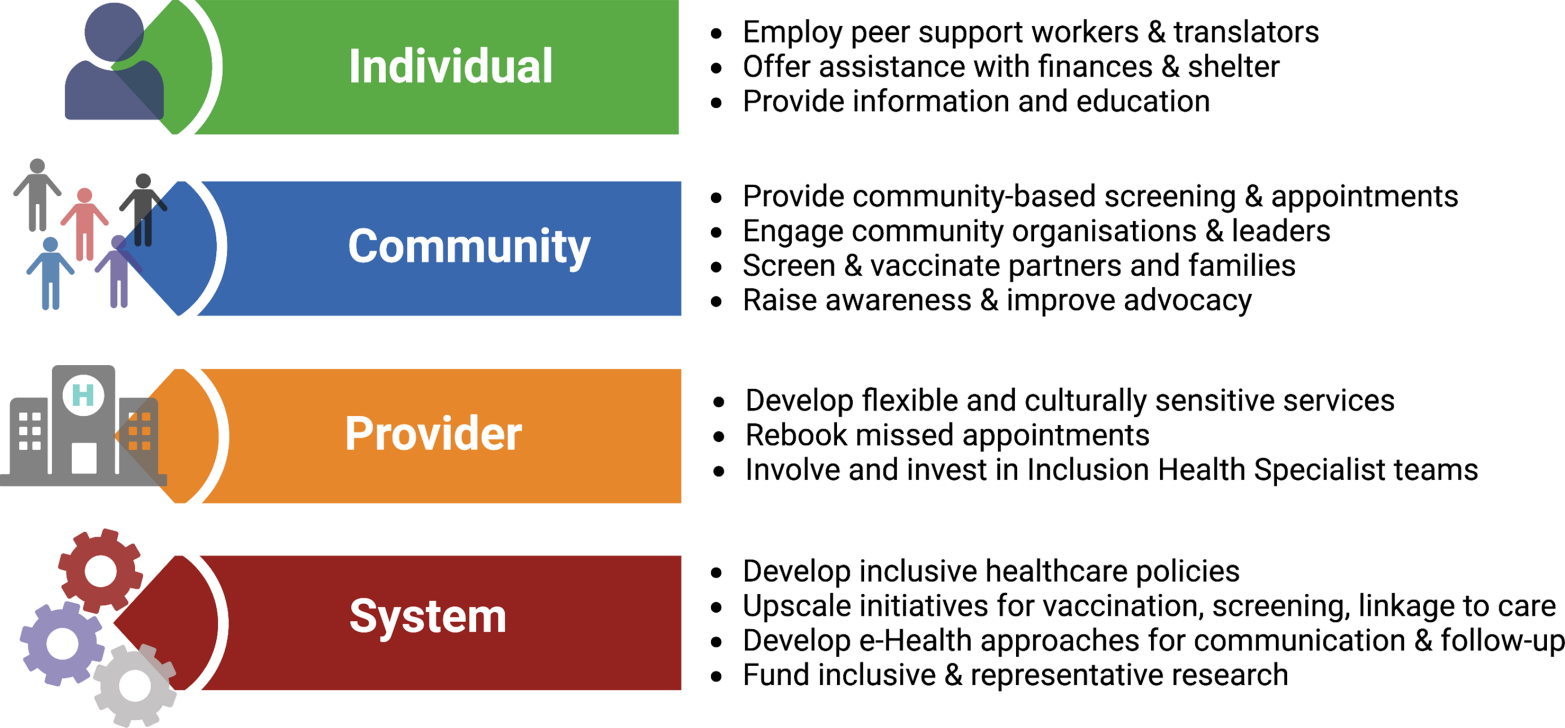
Solutions for service development to overcome barriers for people living with hepatitis B virus (HBV) infection in inclusion health populations, applying framework suggested by Charania et al., 2020. Figure created in BioRender.com with a licence to publish.

Once diagnosed, HBV requires long-term surveillance. However, current models of healthcare fail to provide adequate continuity for inclusion health populations, and innovative solutions are needed. Interventions to reduce the burden of vaccine-preventable infections among migrants can be classified based on the *individual*, *community*, *provider* and *system* (Charania et al., 2020). This framework can be applied to all inclusion health populations (Box 2, Figure 2).

#### Developing holistic approaches

Inclusion health populations face many challenges; therefore, the HBV agenda must not operate in a silo. Integrated infectious disease screening can be cost-effective and an efficient use of resources. An example is the World Health Organization’s ‘triple elimination initiative’ which encourages countries to simultaneously commit to elimination of mother-to-child transmission of HIV, syphilis and HBV (World Health Organization, 2022b). This approach has been demonstrated to reduce disease transmission rates, staff and patient time, and overall programme cost (Zhang et al., 2019).

Integrated infection screening is also recommended in migrants, and in most countries screening tests are offered following a risk assessment (European Centre for Disease Prevention and Control, 2018). Screening is found to be cost-effective, feasible and acceptable, but migrants are a heterogenous group, and services should be culturally appropriate, and accommodate the possibility of fear and stigma surrounding screening and diagnosis (Seedat et al., 2018; Cinardo et al., 2022). In many countries, migrants will undergo pre-entry or port-of-entry screening, missing those who arrive through informal routes (Cinardo et al., 2022). Culturally sensitive outreach services are an important way to reach and screen inclusion health populations (Box 1).

Multi-morbidity is common, for example, incarcerated individuals with HBV have high rates of HIV, HCV and /or TB co-infection (Ahmadi Gharaei et al., 2021; do Nascimento et al., 2020; La Torre et al., 2007), and of substance use and severe mental illness (Kamarulzaman et al., 2016; Fazel and Danesh, 2002). Non-communicable diseases are also prevalent among inclusion health populations (Tweed et al., 2021) for example, PEH have a three-fold elevated risk of cardiovascular disease and cardiovascular mortality compared to non-homeless individuals (Table 2; Al Shakarchi et al., 2020). Moreover, people with co-existing exposures, for example incarceration and substance use, or serious mental illness and substance use, have worse outcomes (Tweed et al., 2021). Diabetes is associated with an increased risk of HBV-associated HCC (hazard ratio 1.36, 95% CI 1.23–1.49)(Campbell et al., 2021). The HBV inclusion health agenda must therefore take a ‘syndemic’ approach, namely to tackle viral hepatitis not as an isolated challenge but recognising its place amongst complex social, physical and mental health challenges and addressing the person as a whole (Mendenhall, 2017; Matthews et al., 2022b).

#### Prioritising patient and public involvement

HBV infection can result in stigma and discrimination, which is magnified when combined with the experiences of socially excluded groups (Matthews et al., 2022a). Sharing lived experiences builds rapport, trust and equality, helping promote advocacy, build peer support networks, navigate healthcare systems and promote diagnosis and treatment of blood borne infections (Paisi et al., 2022; Crone et al., 2022). There is a long history of peer-led approaches with successful outcomes in HIV, TB and HCV (Story et al., 2020; Øgård-Repål et al., 2023). For example, as part of the HepCare project, people with lived experience of HCV infection were upskilled to become equal members of the healthcare team, providing a cost-effective approach to enhancing diagnosis, linkage to care and treatment success (Ward et al., 2019; Surey et al., 2021; Surey et al., 2019). Importantly, a focus group involving people with lived experience ranked advocacy via peer-led services as the second most important intervention behind stable housing (Luchenski et al., 2018). The success and knowledge gained from this peer-led approach must now be translated to HBV.

#### Improving awareness, education, and advocacy

Qualitative research from Australia has identified lack of confidence of healthcare professionals and poor communication surrounding HBV (Wallace et al., 2011; Robotin et al., 2021). Sensitive communication around HBV is of heightened importance in populations who already experience discrimination and stigma, and is a particular consideration where certain subjects are culturally taboo (Condon et al., 2019). Engagement requires sensitivity to cultural nuances, including traditional and religious beliefs (Holmstrom, 2019, Carr et al., 2014). Training for healthcare professionals in trauma-informed care would benefit many inclusion health populations who are more likely to have a history of experiencing assault and torture (Emily, 2021). Lack of awareness is present in healthcare professionals as well as in at-risk populations (Cochrane et al., 2016; Bierhoff et al., 2021). A UK survey of primary care practitioners found many did not believe routine testing of HBV in migrants was necessary, and national guidelines were poorly followed (Evlampidou et al., 2016). Interventions to raise HBV awareness in different groups are paramount to inform action for inclusion health populations.

#### Representative research

Inclusion health populations are often seen as ‘hard to reach’ and may not be viewed as ideal research participants given the possibility of increased loss to follow up and co-morbid conditions. This is further exacerbated in low-resource settings, where there is higher HBV prevalence. There is a striking lack of inclusion health research within Africa, even among global reviews (Table 2). In order to be generalisable and equitable, HBV research needs to be representative of real-world populations and challenges, on a global scale. Performance of new biomarkers, for example hepatitis B core-related antigen, or clinical fibrosis scores, such as APRI and FIB-4, needs to be validated for clinical utility in these populations with overlapping risk factors, multi-morbidity and in varying global populations. While limited treatments currently exist for HBV infection, there is active research into improved treatment and curative therapies; for these to be relevant in populations most affected by HBV, clinical trials must recruit accordingly. Mixed-method approaches should be utilised to understand views and unmet needs of marginalised communities in relation to HBV. The exclusion of inclusion health populations from the current research agenda is likely to further exacerbate inequities when more effective treatments do become available.

### Conclusion

Inclusion health populations comprise diverse groups, but share common experiences including stigma, discrimination, marginalisation, and barriers to accessing diagnostic services and engaging with long-term care. There is a disproportionate impact of HBV among these populations, both caused and exacerbated by social exclusion, which is associated with increased morbidity and mortality. These challenges are even greater in resource-limited settings, where HBV burden is higher but robust data are lacking.

The power and impact of existing studies representing HBV in these populations are limited by small size, cross sectional design and heterogeneity. There are few investigations into interventions, scarce qualitative data, and limited representation of people with lived experience. Moreover, patient advocacy groups for HBV, particularly in inclusion health populations, do not have the same presence or traction as for other infectious diseases, for example TB or HIV. More well-designed prospective studies are needed to allow investigation of the complex interplay between social adversity, co-morbidity and HBV infection to better understand its epidemiology, risk factors, pathogenesis and outcomes within under-served populations. Representative research is crucial to ensure that new discoveries, for example treatments, biomarkers and risk scores, are equitable, relevant, applicable and accessible to the populations where they are most relevant, while qualitative research is needed to understand the perspectives and priorities of the HBV community and inform more effective messaging and advocacy. Innovative methods are urgently required to overcome barriers, adapt healthcare and create services that are fit-for-purpose for inclusion health populations, integrating HBV care into holistic strategies that provide interdisciplinary care to address the complex overlapping needs of inclusion health populations. HBV epitomises the need to adopt a syndemic approach that recognises and addresses the complex interplay between comorbidity and sociocultural contexts.
